# Biomechanical effects of the addition of a precision constraint on a collective load carriage task

**DOI:** 10.1098/rsos.220636

**Published:** 2022-08-24

**Authors:** Nour Sghaier, Guillaume Fumery, Vincent Fourcassié, Nicolas A. Turpin, Pierre Moretto

**Affiliations:** ^1^ Centre de Recherches Sur La Cognition Animale, Centre de Biologie Intégrative, Université de Toulouse, CRCA, UMR CNRS-UPS 5169, 118 Route de Narbonne, 31062 Toulouse, France; ^2^ IRISSE Lab (EA 4075), UFR SHE, Sport Sciences Department (STAPS), Université de La Réunion, 117, rue du général Ailleret, 97430 le Tampon, France

**Keywords:** ergonomics, dual task, load carriage, collective behaviour, team lifting, precision task

## Abstract

Team lifting is a complex and collective motor task comprising motor and cognitive components. The purpose of this research is to investigate how individual and collective performances are impacted during load transport combined with a cognitive task. Ten dyads performed a first condition in which they transported a load (CC), and a second one in which they transported the load while maintaining a ball on its top (PC). The recovery-rate, amplitude and period of the centre-of-mass (COM) trajectory were computed for the system (dyad + table = PACS). We analysed the forces and moments exerted at each joint of the upper limbs of the participants. We observed a decrease in the overall performance of the dyads during PC: (i) the velocity and amplitude of CoM_PACS_ decreased by 1.7% and 5.8%, respectively, (ii) inter-participant variability of the Moment-Cost-Function and recovery rate decreased by 95%, and 19.2%, respectively during PC. Kinetic synergy analysis showed that the participants reorganized their coordination in the PC. We demonstrated that adding a precision task affects the economy of collective load carriage at the PACS level while the upper-limbs joint moments were better balanced across the paired participants for the PC.

## 1. Introduction

Individual manual material handling is commonly performed in many human activities. Its definition encompasses essentially different tasks of load handling (i.e. transport, support, lift, push, pull…) and has unfavourable ergonomic conditions (Directive 90/269/CEE). Different lifting techniques [1] and mechanical aids [2] were proposed to avoid the associated leisure. However, the most common alternative used is team lifting. It is, in appearance, a simple solution to carry a heavy load (i.e. too heavy to be carried safely by a single individual), a bulky object without mechanical aid. This strategy is therefore supposed to reduce the load on individuals performing the task [3,4]. It is also used in some sports such as Crossfit discipline when lifting the ‘worm’, a heavy, long and soft cylindrical bag [5].

Furthermore, team lifting necessitates both motor and cognitive skills in order to control movement coordination. In fact, the achievement of this task requires a set of cognitive actions in order to deal with the actual situation, to fine-tune the response using feedback, to monitor performance, and to inhibit task-irrelevant information [6]. For this reason, many authors consider it as a dual task, e.g. ironwork [7]. The tasks are called dual as they often undergo some interference, linked to a limited ability to share attention between the two task goals. Dual-task interference is commonly studied in psychology to highlight the cognitive limits of the human brain [8]. One well-known example of these cognitive limits is based on locomotion which can be used in the interference paradigm [9,10]. For example, walking along an L-shaped path while performing an arithmetic task deteriorates the mobility function. Any additional cognitive task is considered as a limiting factor to the motor task since it induces a modification of the gait pattern, e.g. reducing gait speed, inducing movement fluctuation and oscillation. Beach *et al.* [11], showed that during a repetitive lifting task adding a precision placement challenge leads to an increase in lumbar spine load and an increase in the upper limb movement time.

So far, no consensus has been reached to evaluate gait modification due to load addition. Recent studies focused on lifting-precision dual-task and showed that locomotor pattern was not affected when the participants performed a dual-task such as carrying a load (20% of mean body mass) on their shoulders [12], or carrying a load (21% and 36% of mean body mass) on their backs [13,14]. However, other studies reported a walking pattern affected by the dual-task when the load is balanced on the top of the head [15] or when it is carried collectively [16]. In certain jobs, a collective load transfer is simultaneously associated with a precision task (e.g. talking with the patient in nursing practice administrating medication in stretchers bearing, industrial manufacturing). This occurs especially during search and rescue or paramedics activities [17–20].

Despite this practical relevance, only a few studies deal with the biomechanical aspects of the collective transportation of a load. However, it is essential to conceive aids, exoskeletons and collaborative robots dedicated to assisting humans in such tasks. Fumery *et al*. [16] studied the energetic exchanges taking place during a collective load carriage to investigate whether two individuals transporting an object behave economically. The authors showed that the external energetic exchanges occurring during this type of transport were as efficient as those occurring in single gait when the load is below 10% of the total body mass of the dyad. In our study, we reproduce the protocol of Fumery *et al*. [16] to investigate the locomotor pattern of ten paired participants carrying a box collectively, and to compare these walking patterns when the task is performed simultaneously with a precision task. The precision task consists in maintaining a ball at the centre of a circular target drawn on the top of the box and provides insight into the dyad motor collaboration.

Thanks to handle sensors and kinematic data, we also record the forces and moments applied to the box and then estimate the constraints at the arms and back joints using the inverse dynamics bottom-up procedure. The purpose of this study is to investigate how individual and collective performances are impacted during load transport when combined with a cognitive task, and in what way the control of the cognitive task is shared across the participants. Since it has been observed in single participants walking and performing a cognitive task, we hypothesized that participant's gait performance of walking while collectively transporting a load is disturbed when they perform a dual-task requiring precision. In fact, we expect that the precision task compels the dyad participants to modify their behaviour in order to comply with the ball control, leading to a decrease of the pendulum-like behaviour of the centre of mass (CoM), an increase in the muscular effort, and the modification of the synergies inter-participant necessary to perform the task. Fumery *et al*. demonstrated that the pattern of a dyad carrying a light load was not affected by the collective task; our hypothesis is therefore that, using the same load ratio, the precision task is the single factor which affects the walking-carrying pattern and the inter-participants synergies of the paired participants [16].

## 2. Material and methods

### 2.1. Population

Ten pairs of healthy male participants (mean ± s.d.: volunteer 1—at the left side of the load: height = 1.77 ± 0.07 m, mass = 74.78 ± 9.00 kg; volunteer 2—at the right side of the load: height = 1.77 ± 0.05 m, mass = 74.54 ± 12.38 kg) took part in the experiments. The participants had no orthopedic disabilities, no dysfunctions of the locomotor system, no neurological or vestibular diseases, no visual deficits and no proprioceptive disorders or dementia.

This study was carried out in accordance with the requirement of a non-interventional study given by the CNRS bioethical office. The study was approved by the Research Ethics Committee of the University of Toulouse, France (number IRB00011835-2019-11-26-172, Université Fédérale de Toulouse IRB #1). All participants gave verbal and written informed consent in accordance with the Declaration of Helsinki.

### 2.2. Experimental protocol

Two conditions were performed by the participants. In the first condition (Control Condition: CC) they walked side by side at spontaneous speed while carrying a box (mass = 13.41 kg, size: 0.40 × 0.40 × 0.28 m) equipped with two lateral handle sensors (Sensix, France). The mass of the box plus the sensors was 14.250 kg thus almost 10% of the body mass of the two volunteers. In order to get accustomed to the task, the participants performed three successive trials. Only the third trial was kept for the analysis.

**Figure 1.**
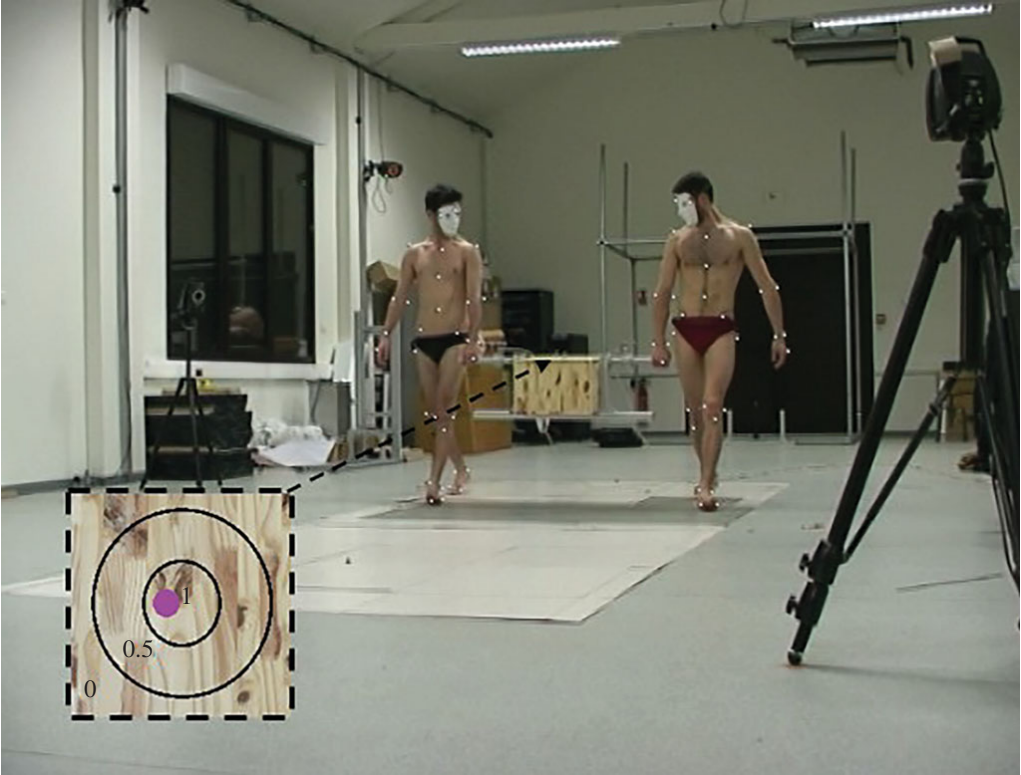
experimental setup: collective load carriage performed with a precision task (PC).

In the second condition (Precision Condition: PC), the participants were instructed to transport the box while performing an accuracy task consisting of keeping a ball (diameter: 19 mm, mass: 2 g) at the centre of a circular target drawn on the top of the box (figure 1). Participants were not allowed to orally communicate together during the experiments.

### 2.3. Kinematic and kinetic data acquisition

Motion capture data were collected using 13 infrareds (11 MX3 and 2 TS40) transmitter-receiver video cameras (Vicon, Oxford metric's, Oxford, United Kingdom) sampled at 200 Hz. Forty-two retro-reflective markers were placed on bony landmarks and on the navel of each participant, and 14 on the box. The ball used during the PC tests was reflective as well and was tracked by the Vicon system [21,22].

In order to record the gait pattern at constant speed (i.e. to exclude the acceleration and deceleration phases at the beginning and end of each trial) the volume calibrated by the Vicon system (30 m^3^) was located in the middle of the 20 m-long walkway crossed by the participants. The reflective marks were tracked to define the kinematics of the Poly-Articulated Collective System (PACS) formed by the two participants and the load they carry [23,24]. The data were recorded and investigated for only one gait cycle. This gait cycle is defined by the first heel strike of the first participant and the third heel strike of the second participant in order to ensure a gait cycle of each participant. The three-dimensional reconstruction was performed using Vicon Nexus 1.8.5 software.

The two lateral handles used to transport the box were equipped with Sensix force sensors sampled at 2000 Hz. A 4th order Butterworth filter and a 5 Hz and 10 Hz cut frequency were applied to analyze the positions of the markers and the forces exerted on the box handles, respectively.

### 2.4. Computed parameters

#### 2.4.1. CoM_PACS_ trajectory

The De Leva Anthropometric tables [25] were used to estimate the mass *m*_*i*_ and the CoM of each segment *i* (CoM_i_) of the PACS and to compute its global CoM (CoM_PACS_) as follows:

2.1 $$G_{\mathrm{PACS}}=\frac{1}{m_{\mathrm{PACS}}}\overset{n=33}{\underset{i=1}{\sum }}m_{i}G_{i},$$

with *G*_PACS_ the three-dimensional position of the CoM_PACS_ in the frame R (the global coordinate system), *m*_PACS_ the mass of the PACS, *n* the number of PACS segments (i.e. 16 segments per volunteer plus one segment for the box) and *G_i_* the three-dimensional position of the CoM*_i_* in the frame R. The CoM of the box was determined at the intersection point of the vertical lines obtained by hanging it with a thread fixed at different positions. The material used for the box construction, i.e. wood and aluminium, was considered as not deformable.

According to Winter, the amplitude (A = *Z*max – *Z*min, with *Z* the height of the CoM_PACS_, in meters,) and the period (peak to peak, in percent of the gait cycle) of the CoM_PACS_ were also assessed [26].

The forward kinetic (Wkf), as well as the vertical (Wv) and external work (Wext) of the CoM_PACS_ were computed according to the method of Bastien *et al*. [14]. Then based on the external work, the percentage of energy recovered of the CoM_PACS_ in the sagittal plane was computed (called recovery rate RR in [16,27]). This parameter assesses the amount of energy transferred between the potential and the kinetic energy (equation (2.2)),

2.2 $$\mathrm{RR}=100\frac{Wkf+Wv-Wext}{Wkf+Wv}.$$

The closer the value of RR to 100%, the more consistent the locomotor pattern is with the inverted pendulum system (IPS) model of locomotion [14,28–30]. In this study, we investigated the evolution of the CoM_PACS_ trajectory.

#### 2.4.2. Forces and moments at the joints of the upper limbs

Sensix force sensors recorded the forces and moments applied by each participant to the two box handles. Before the computation, the data of the sensors located by specific markers were transferred to the Galilean frame of the laboratory using rotation matrix. A cross correlation method was applied in order to analyze the coordination between the forces produced by both participants. To investigate whether the movement of the box results from an action-reaction strategy, we computed the time lag required for the position of the left side and right side of the box to be the same on the medio-lateral, antero-posterior and vertical axis in CC and PC. The coordination was assessed through the forces exerted in three directions (medio-lateral, antero-posterior and vertical axis). These results will reflect the level of coordination of two participants during a collective transport.

In order to quantify muscular constraints produced at the upper limb, the Inverse Dynamic Method was used to estimate forces and moments at each joint of the upper limb. The Moment Cost Function was then computed (kg. m^2^. s^−2^, [31]) as follows:

2.3 $$\mathrm{MCF}=\sqrt{M_{L_{wt}}^{2}}+\sqrt{M_{R_{wt}}^{2}}+\sqrt{M_{L_{el}}^{2}}+\sqrt{M_{R_{el}}^{2}}+\sqrt{M_{L_{sh}}^{2}}+\sqrt{M_{R_{sh}}^{2}}+\sqrt{M_{\mathrm{back}}^{2}}+\sqrt{M_{\mathrm{neck}}^{2}},$$

where *M*_*L_wt*_, *M*_*R_wt*_, *M*_*L_el*_, *M*_R_el_, *M*_*L_sh*_, *M*_*R_sh*_, *M*_back_ and *M*_neck_ are the mean values over a PACS gait cycle of the three-dimensional left and right wrist, left and right elbow, left and right shoulder, top of the back and neck moments, respectively. $\sqrt{M^{2}}$ represents the Euclidean norm of *M* (i.e. $\sqrt{M^{2}}=\sqrt{\sum_{i=1}^{3}{\left(M_{i}\right)}^{2}}$ with *Mi* the i-th component of the vector *M*).

Then, the MCF values of each participant were summed up to obtain the total moment cost function (Total MCF). This total MCF allows us to quantify the global effort produced at the upper limbs of the PACS during one gait cycle. Finally, the MCF difference (Δ MCF) was computed as the difference between the two individuals to investigate whether the participants produced the same effort in the upper limbs during the load transport.

#### 2.4.3. Kinetic synergy analysis

We extracted the kinetic synergies by using a principal component analysis (PCA) applied to the wrist, elbow, shoulder, back and neck joint moment on the right and left sides of the body. The y-component of the joint moments data from one trial per condition was arranged in time × joint moment matrices. The joint moments were normalized by their amplitude and centred before application of the PCA. VAF (Variance Accounted For) corresponded to the cumulative sum of the eigenvalues normalized by the total variance (the sum of all eigenvalues). The synergy vectors were rotated using Varimax rotation to improve interpretability.

We first extracted the synergy vectors for each experimental condition and each participant separately. In this analysis, the initial data matrices were constituted of all available time frames in line, concatenated from one trial per condition, and of eight columns corresponding to each joint moment, namely the right wrist, left wrist, right elbow, left elbow, right shoulder, left shoulder, back and neck. Based on a previous study we extracted three synergies in this analysis; this method consisted in selecting the first number allowing to reach a VAF ≥ 90%. We then performed a second analysis to identify possible co-variations between the joint moments of the two participants in each pair. The columns of the initial matrices were thus constituted of the joint moments of the two loaded arms, i.e. the right wrist, elbow and shoulder joint moments of participant #1, plus the left wrist, elbow and shoulder joint moments of participant #2. Based on a previous study we extracted two synergies in this analysis. We used Pearson's *r* to order the different synergies similarly across the different participants and conditions.

#### 2.4.4. Accuracy score

A performance score (Score_p_) was assigned to each image of the videos captured by the Vicon system (200 images/s). The score depended on the location of the ball in the target: 1 when the ball was inside the small circle, 0.5 when it was between the small and the large circle and 0 when it was outside the large circle. The accuracy over the whole gait cycle was measured by an overall score (Score_accuracy_), expressed in percentage, and calculated as follows:

4 $${\mathrm{Score}}_{\mathrm{accuracy}}=\frac{\sum {\mathrm{Score}}_{p}\times 100}{t_{\mathrm{gait}\;\mathrm{cycle}}},$$

where *t*_gait cycle_ represents the number of Vicon images recorded along one gait cycle.

#### 2.4.5. Orientation of the upper part of the body

The head, shoulders and pelvis rotation angles were computed around the vertical axis of each participant in the two conditions. The angle was positive when the participants turned towards the box they carried; otherwise, it was negative. The distance between the forehead and the sternum (distance FOR-STE) was also computed in order to investigate the flexion of the cervical spine.

### 2.5. Data analysis

The data were analysed with Matlab R2016b and StatView 5.0 software. A paired *t*-test was used to compare the RRs, the amplitudes, the periods, the velocities of the vertical displacement of the CoMPACS, the head, shoulders and pelvis rotation angle and the length FOR-STE between the CC and PC condition. The significance threshold was set to 0.05. We also performed a Fisher Z-transformation in order to get normally distributed correlation coefficient*.* Using a cross-correlation method we computed the time lag required for the position of the left side and right side of the box to be the same on the medio-lateral, antero-posterior and vertical axes in CC and PC.

We used the average subspaces angles to compare the subspaces spanned by the synergy vectors [32]. In order to decide whether the subspaces were more similar than expected, the confidence interval (CI) of random comparisons was computed. For this analysis, we generated pairs of random subspaces constituting each of three unit vectors of dimension 8 (individual PCA analysis) or,two unit vectors of dimension 6 (conjoint PCA analysis) and computed the mean subspace angle between them. The unit vectors were built using normally distributed pseudo-random numbers (Matlab randn function). We performed 10 000 simulations in order to determine the 95%-CI of the mean subspace angle between the pairs of random subspaces. The confidence interval was 39.5°–70.0° (55.0 ± 7.6°) for the individual PCA analysis and 36.3°–79.1° (57.7 ± 10.7°) for the conjoint analysis.

We used Student's tests for single mean to compare the subspaces angles to the lower bound CI with the assumption that similarity was higher than expected by chance when the angles were lower than the lower bound CI.

VAFs were compared with an ANOVA with one repeated measure (control versus precision conditions) and one factor (participant #1 versus participant #2) when synergies were extracted separately for each participant. For the conjoint analysis, a paired Student *t*-test was used. Subspaces angles were compared with *t*-tests for dependent samples (paired *t*-test) when comparing the control and precision conditions, and *t*-test for independent samples when comparing the two participants. Adjustments for multiple comparisons were performed by Bonferroni's method. Initial level of significance was set to *p* < 0.05.

## 3. Results

### 3.1. Dynamics analysis of the dual-task

#### 3.1.1. CoM_PACS_ trajectory

**Figure 2.**
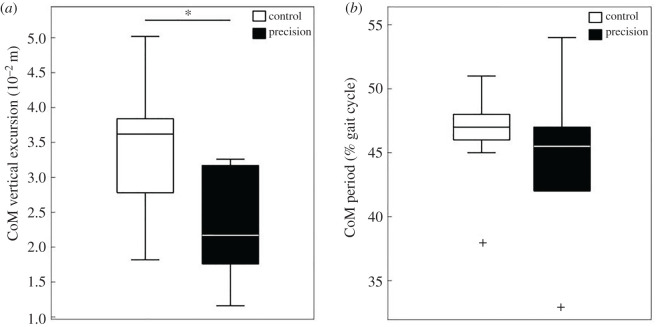
Boxplot of the vertical excursion (*a*) and period (*b*) of CoM_PACS_ in Control Condition (CC) and Precision Condition (PC).

The CoM_PACS_ velocity significantly decreased from 1.40 ± 0.14 m s^−1^ in CC to 1.23 ± 0.17 m s^−1^ in PC (*t* = 3.385, *p* = 0.008). The CoM_PACS_ amplitude (figure 2*a*, *t* = 3.704, *p* = 0.005), significantly decreased from 2.87 ± 0.742 cm in CC to 2.29 ± 0.739 cm in PC (electronic supplementary material, table S3). However, the period of the CoM_PACS_ oscillation was not significantly affected by the precision task (figure 2*b*, *t* = 0.842, *p* = 0.422).

**Figure 3.**
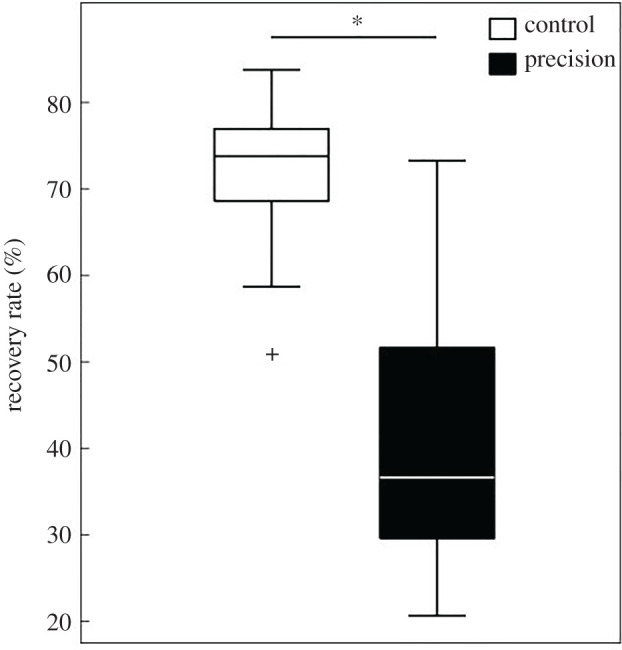
Boxplot of the recovery rate (%) boxplot of the vertical excursion (*a*) and period (*b*) of CoM_PACS_ in control condition (CC) and precision condition (PC).

The percentage of energy recovered at the CoM_PACS_ significantly decreased between CC and PC (*t* = 5.18, *p* < 0.001) (figure 3). This showed an alteration in the efficiency of the locomotor pattern of the dyad when the energy transfer between the potential and the kinetic energy was 19.2% lower in PC compared to CC.

#### 3.1.2. Forces applied to the handles

**Figure 4.**
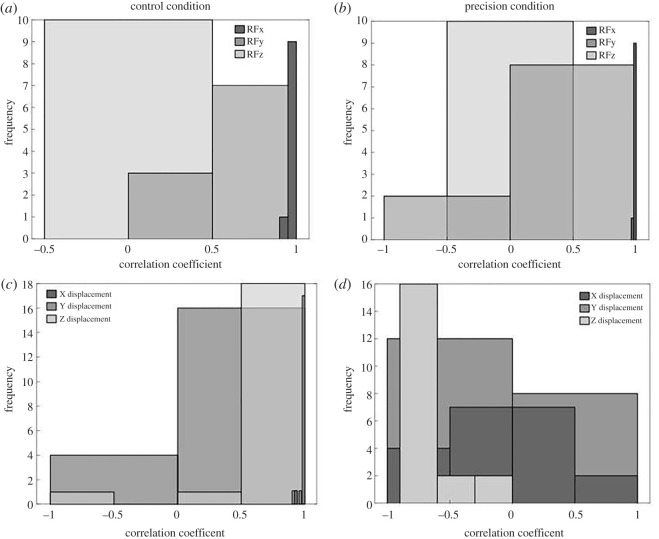
Histograms of correlation coefficient (COR) distribution. (*a,b*) COR of the forces produced by the participant in each dyad on the box handles, on the medio-lateral (Fx), antero-posterior (Fy) and vertical axis (Fz) in the CC (*a*) and PC conditions (*b*). (*c*) Cor of the ball, and handles displacement, on Fx, Fy and Fz, in the CC and PC conditions. (*d*) Cor of the ball trajectory and the sum of the participant exerted forces on the handles, on Fx, Fy and Fz, in PC. *0.05 > *p >* 0.01; **p < 0.01.

Significant differences were found between the correlation coefficients of the three components (x, y, z) of the forces applied to the handles by the participants for each condition. Forces applied to the handles differ according to the condition. In CC, RFy was lower than RFx and RFz, the same results were found for PC (Statistical results in supplementary). Comparison of CC and PC correlation coefficients shows significant difference for the medio-lateral axis (RFx) and the vertical axis (RFz). In fact, there is a higher correlation coefficient RFx (*p* < 0.05) and lower correlation coefficient RFz (*p* < 0.05) during PC (figure 4).

In the CC, time lags were lower than 150 ms in the medio-lateral and antero-posterior axis and only one lag was higher than 150 ms for one dyad in the vertical axis (table 1, lagZ 20 ± 50 ms). Concerning the PC, the time lags were also lower than 150 ms in the medio-lateral and antero-posterior axis, and five dyads had a time lag higher than 150 ms in the vertical axis (table 1, lagZ 180 ± 230 ms) (electronic supplementary material, table S7).

**Table 1.** The action-reaction strategy, the time lag (s) required for the position of the left side and right side of the box to be the same on the medio-lateral, antero-posterior and vertical axis in CC and PC. * 150 ms < *p* [33].

group	control condition			precision condition		
	LagX	LagY	LagZ	LagX	LagY	LagZ
1	0	0	0	0	0	0,155*
2	0	0	0,04	0	0	0,56*
3	0	0	0	0	0	0
4	0	0	0	0	0	0
5	0	0	0	0	0	0
6	0	0	0	0	0	0,15*
7	0	0	0,15*	0	0	0
8	0	0	0	0	0	0,355*
9	0	0	0	0	0	0
10	0	0	0	0	0	0,545*

#### 3.1.3. Dynamic synergy analysis

Consistent with a previous study we extracted three dynamic synergies for all participants, which accounted for 96.3 ± 2.0% of total variance on average (range [90.0–99.0] %). We found no effect from the side (being on the left or right side of the load) nor from the precision constraint on the VAF values (i.e. |ΔVAF|= 1.9 ± 0.4%, *p*-value = 0.24, *η*² = 0.08 and |ΔVAF|= 0.9 ± 0.5%, *p*-value = 0.62, *η*² = 0.01, respectively).

**Figure 5.**
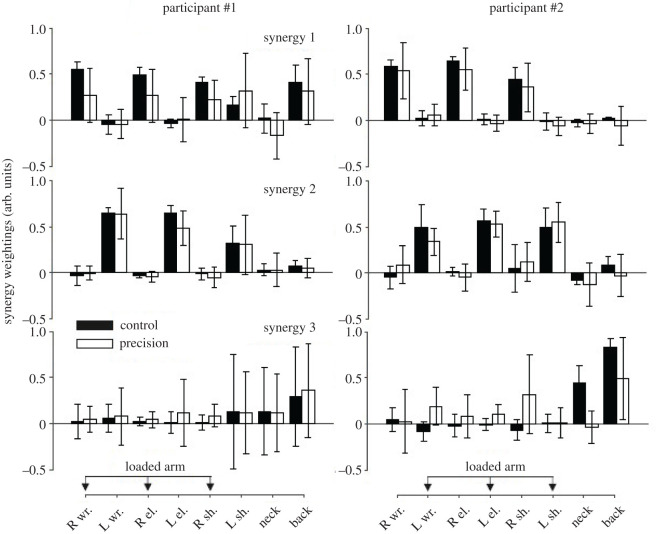
Dynamic synergy vectors. Three synergies accounted for more than 90.0% of total variance in all participants.

**Figure 6.**
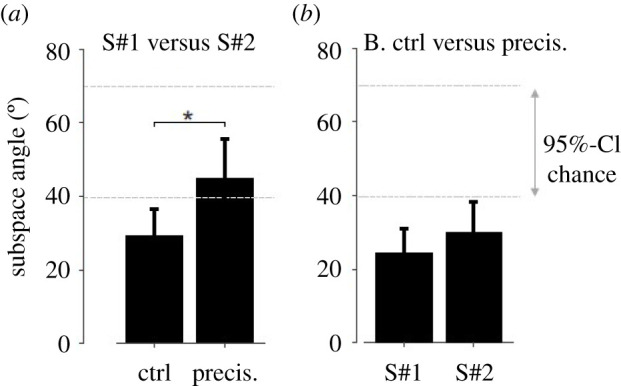
Subspaces comparison. The subspace angle measures the similarity between the subspaces spanned by the dynamic synergies. The 95%-confidence interval of angles obtained with random synergies (95%-CI chance) is indicated, i.e. CI = [49.5°, 70.0°]. *p < 0.001.

The dynamic synergies for each participant are depicted in figure 5. The comparisons between participant #1 and participant #2 gave subspaces angles that did not differ from those expected by chance in the precision condition (i.e. 45.0 ± 10.0° compared to 39.5°, Student *t*_9_ = −1.34; *p*-value = 0.21; figure 6*a*). For the other comparisons in figure 6, subspace angles were lower than expected by chance (figure 6, |Student *t*_9_ ≥ 7.6; *p*-value < 0.001). The subspace angles were lower in the control condition than in the precision condition when comparing participant #1 and participant #2 (figure 6*a*, Cohen's *d* = 1.9; Student *t*_9_ = −4.95; *p*-value < 0.001), showing an effect of the conditions on inter-participant similarities. The comparison between the control and precision conditions gave subspace angles of 27.2 ± 5.7° on average, with no differences between participants (figure 6*b*, Cohen's *d* = 0.61; Student *t*_9_ = −1.93; *p*-value = 0.09). The inter-condition subspace angles were lower than inter-participant subspace angles (i.e. 27.2 ± 5.7° versus 37.1 ± 7.7°, Cohen's *d* = 1.5; Student *t*_18_ = 3.30; *p*-value = 0.004).

**Figure 7.**
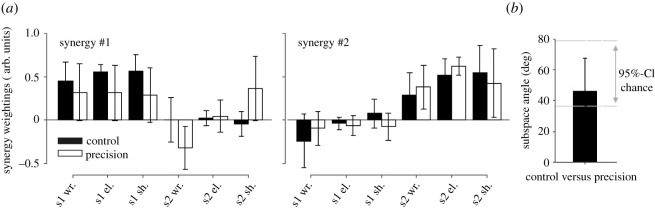
Conjoint synergies. S1 and S2 refer to participants #1 (right side) and participant #2 (left side), respectively. The 95%-confidence interval of angles obtained with random synergies (95%-CI chance) is indicated in panel (*b*), i.e. CI = [36.3°,79.1°].

For the conjoint PCA analysis, two dynamic synergies were extracted for all pairs of participants, which accounted for 95.1 ± 3.5% of total variance on average (range [84.4–98.8]%). The dynamic synergies for the conjoint analysis are depicted in figure 7. VAFs were similar in the control and precision conditions (96.6 ± 2.5% versus 93.5 ± 3.8%, respectively, Cohen's *d* = 0.7; Student *t*_9_ = 2.18; *p*-value = 0.06). The subspace angles were not different than expected by chance (i.e. 46.4 ± 21.3°compared to 36.3°, Student *t*_9_ = 1.50; *p*-value = 0.17) when comparing the control and precision conditions (figure 7*b*).

#### 3.1.4. Moment cost function (MCF)

**Figure 8.**
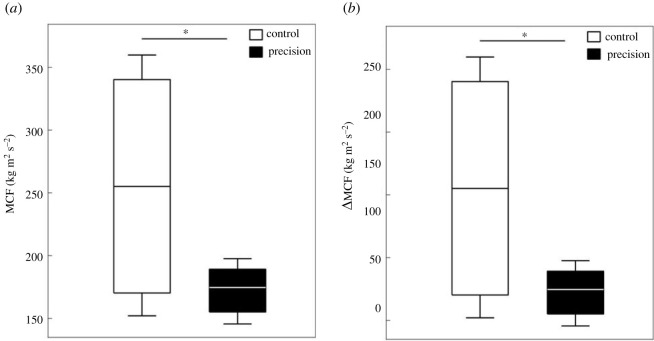
Boxplot of PACS MCF (*a*) and ΔMCF (*b*) in the Control Condition (CC) and the Precision Condition (PC). * = significant difference (*p* < 0.05 paired *t*-test).

In the CC the results obtained were divided into two different groups of dyads. Five dyads showed higher values in both total MCF (figure 8*a*), (33 935 kg m^2^ s^−2^ ± 20,44) and ΔMCF (figure 8*b*), while five other dyads showed lower values, (16 917 kg m^2^ s^−2^ ± 17,06). The results of total and *Δ*MCF (respectively 17 266 kg m^2^ s^−2^ ± 19,16; 5,78 kg m^2^ s^−2^ ± 12,85) varied less in PC than in CC.

### 3.2. Added task

#### 3.2.1. Accuracy score based on the ball trajectory

Regarding the ball trajectory, we found a correlation (figure 4*c*) between the displacement of the handle and the ball on the X-medio-lateral axis (P1 = 0.608 ± 0.62; P2 = 0.606 ± 0.62). The same goes for the displacement on the Y-axis which represents the postero-anterior axis (P1 = 0.999 ± 0.001; P2 = 0.999 ± 0.001). On the vertical Z-axis, the ball displacement and the handle are positively correlated (P1 = 0.699 ± 0.498; P2 = 0.785 ± 0.224).

Correlations were also computed in order to study the relationship between the ball trajectory and the sum of forces exerted by the participants on the handles (figure 4*d*). Only two of ten dyads had a correlation on the X-axis; a positive correlation for dyad 1 and a negative correlation for dyad 10 (P1 = −0.034 ± 0.411; P2 = −0.167 ± 0.471). On the Y-axis five inten dyads had a significant negative correlation compared to the other three dyads who had a significant positive correlation (P1 = −0.182 ± 0.659; P2 = −0.162 ± 0.611). For the vertical Z-axis, nine in ten dyads had a positive significant correlation (P1 = −0.724 ± 0.115; P2 = −0.62 ± 0.262).

#### 3.2.2. Accuracy score

The mean Score_accuracy_ was 80.45 ± 23.66% during one gait cycle of the PACS in the PC condition.

### 3.3. Head and trunk

Table 2 shows that when participants had to keep the ball in the centre of the target they turned the upper part of their body toward the box. Indeed, across the CC and the PC condition the orientation towards the box increased by 57.42, 9.22 and 3.29 degrees for the head, shoulders and pelvis, respectively. Also, the distance FOR-STE decreased by 7.69 cm between the CC and PC conditions, showing that the participants were gazing at the box (electronic supplementary material, table S5).

**Table 2.** Head, shoulders and pelvis orientation (°) and distance between the forehead and the sternum (cm) in the CC and PC. *0.05 > *p* > 0.01; **p < 0.01.

	CC	s.d.	PC	s.d.
head orientation	2.58	±4.61	60.00**	±11.81
shoulders orientation	1.99	±3.13	11.21**	±6.61
pelvis orientation	0.16	±5.14	3.45*	±4.05
distance FOR-STE	21.67	±4.86	13.98**	±3.25

## 4. Discussion

In this experiment, 10 dyads of participants transported a load in two different conditions: a Control Condition (CC), in which they walked together transporting a load, and a Precision Condition (PC), in which they walked together transporting a load and maintaining at the same time a ball on its top. The first objective of our study is to investigate how individual and collective performance is impacted during load transport when combined with a cognitive task, and in what way the control of the cognitive task is shared across the participants

We studied the Centre of Mass (CoM) of the system formed by paired participants with the box they carried. The result showed that the CoM_PACS_ speed decreased when the precision constraint was added. Besides, the second task induced a decrease in the pendular behaviour and amplitude of the system. However, the period of the CoM_PACS_ displacement was not affected in PC. These results could be expected as the added task can be considered as a fine motor skill [34]. Indeed, and as observed in the gait of an adult performing a dual-task [10], the individuals needed to reduce their speed to perform both tasks. Similar to the findings of Holt *et al*. [35] in a single carrier, the decrease in speed was accompanied by a decrease in the CoM_PACS_ vertical amplitude. These adaptations are reminiscent of the classic speed-accuracy trade-off, and the reduction in speed is very likely a strategy to reduce motor noise and improve controllability.

To explore the task further, we also studied each participant as a distinct entity. The comparison of the individual CoM trajectory of both participants for CC and PC did not show any significant difference. Indeed, when walking side-by-side with a sensory interaction, participants tend to synchronize their walking pace and kinematics [36,37]. The same goes for the CoM parameters (velocity, and CoM amplitude), i.e. no significant differences were found between the participants on the left and the ones on the right. The comparison of joint angles showed only a significant difference in hip angles between participants.

No matter what condition they performed, the participants tend to synchronize their speed and gesture frequency. However, the precision task altered the CoM_PACS_ kinematics leading to a less efficient energy transfer. In fact, this spatio-temporal strategy induced a decrease in the pendulum-like behaviour at the CoM_PACS_. Here, the energy recovered (RR) values obtained for the PACS in the CC (mean ± CI 0.95 = 60.25 ± 8.57%) were similar to those obtained in single carriers alone, as measured by Bastien *et al*. in Nepalese porters and in untrained individuals (RR = 61%) [14], or by Tesio *et al*. in healthy individuals (RR = 60%) [38]. Our results showed a significant RR decrease in PC. This confirms the CoM_PACS_ pendulum-like behaviour alteration. The potential and kinetic energy being out of phase, RR decrease leads to a higher mechanical cost for the whole system due to the precision task.

Research on collective tasks showed that when sharing visual information on their performance, group members tend to coordinate their forces and movements [36,39,40]. Hence, the visual feedback could have an impact on the muscular effort variability between dyads, as well as between participants within each dyad. The global muscular efforts estimated thanks to the moment cost function (MCF) of the upper limbs were much more shared across the individuals of each dyad when they performed the dual-task than in the lifting condition (figure 8). The decrease of the MCF variability across the dyads demonstrated a convergence of the upper limb kinematic and kinetic data due to the constraint (figure 8). It revealed a more shared and common motor control across the participants in response to the dual-task. But, both adaptations revealed by the MCF and *Δ*MCF lead to a lower cost of the upper limb contribution to the precision task that was unexpected.

Doi *et al*. demonstrated that increasing the difficulty of a task (e.g. dual-task) affects the cost of the movement in elderly adults [41]. Similarly, we found a modification in the trunk posture in PC that might have resulted in a finer control of the task. During PC, participants spontaneously oriented their head, shoulders and pelvis toward the box, to gather more visual information. Modification of the upper body orientation seems to allow the participants to look at the ball on the top of the load but, at the same time, these body segments were locked in a position that likely disturbed the kinematic of the PACS and their ability to behave like a pendulum. This interpretation is in accordance with the findings of Winter, who suggested that during bipedal walking, the control of the trunk restrains vision and head control [26]. Here, the swings and rotations of these segments (head, shoulders and pelvis), while locked during a gait cycle, do not contribute to the CoM_PACS_-evolution to time but may explain the lower pendulum-like behaviour in the PC. The decrease of the vertical amplitude of the CoM_PACS_ with both trunk and head fixed to look at the ball reveals a lower limb pattern altered in PC. Numerous research studied the impact of the trunk posture on gait pattern, whether for medical purposes [42] or sport performance purposes [43,44]. These studies showed that a modification of trunk posture on the frontal and sagittal plane influences the bilateral lower limb kinematics and muscle activity. Here, the control of the walking speed and of the ball may have been supported by the lower legs and induced the dissipation of the mechanical energy thanks to eccentric work of the muscles. This would have been quantified thanks to inverse dynamic at the lower limbs. Unfortunately, it was not possible to record the ground reaction forces of both participants at the same time.

The impact of the added task on the physical action of the participants during the load transport was investigated. We recorded the forces applied by the participants to the two box handles (figure 4) during CC and PC. The participants' coordination was investigated through the correlation coefficient of applied forces. The results showed, in both conditions, a positive correlation for the forces on the antero-posterior and vertical axis. However, the correlation on the medio-lateral axis was weaker, with a large variability across the dyads of individuals. Thus, it seemed that the participants coordinated their forces to move the load in the up-down and forward directions, without adopting a common strategy for the left-right direction. The results were similar between the two conditions, showing that the second task did not affect the collective strategies used during a simple load carriage task.

We considered that an action-reaction strategy was involved when the lag was higher than 150 ms, which corresponds to the minimum latency observed to take a decision after the perception of a stimulus [33]. All lags were lower than 150 ms in the medio-lateral and antero-posterior axis. On the vertical axis, however, lags higher than 150 ms were found in one dyad in the CC and in five dyads in the PC. Therefore, a modification of the behaviour for half of the dyads was observed when the second task, requiring accuracy and precision, was added. These results might suggest a more conscious control of the box. It seems that the participants moved the ball essentially by applying a combination of force and moment inducing a torque around the vertical axis, the rotation of the box around the anteroposterior axis, and the displacement of the ball along the slope. When the box was thus moved by one participant, the second participant reacted (with a reaction time >150 ms) by moving it in a different direction in order to keep the ball in the centre of the target.

Our results also suggest that the load carriage was affected by the second task, independently of the participants' performances in this task. Indeed, the accuracy score was not correlated (*r* = 0.31, *p* = 0.386) to the RR. The accuracy score was high, suggesting a good investment from the participants in the second task. According to Yogev-Seligmann *et al*. walking is a complex motor activity, which requires both the mobilization of executive functions, i.e. the cognitive capacities that allow an immediate adaptation of the motor behaviour, and precision [9]. On one hand, the decrease in locomotor performance in the precision condition could be explained by an increase in precision and a decrease in the mobilization of the executive functions used in locomotion. On the other hand, it also could be explained by a strategy of prioritization due to a structural interference between the precision needed to realize the first and the second task. Ebersbach *et al*. concluded that even when a task is highly practiced (e.g. walking), adding concurrent tasks would lead to strategy changes depending on the attentional demand [45]. Indeed, these control strategies are commonly used for humanoid robots when generating movement prioritization. Sentis and Khatib proposed a multi-level control hierarchy where the global task is decomposed into several subtasks [46]. The hierarchy used ensured that constraints and critical tasks were accomplished first while optimizing the execution of the global task. However, the absence of correlation between the accuracy score and the RR value reveals that the precision task may have been too easy and may not discriminate between different levels of precision.

Concerning the kinetic synergy analysis, a first observation is the non-symmetry in terms of vector weightings for the loaded and unloaded arms. For example, the wrist, elbow, and shoulder joint moments covary more with the neck and back joint moments for the loaded arm than for the unloaded arm, as demonstrated by high weightings coefficients for these joints (figure 5). Participants used more similar synergies during the CC than during the PC (figure 6*a*). The conjoint synergies were less similar than expected when comparing the control and precision conditions (figure 7*b*). These two results show that a change in inter-joint moments coordination occurred due to the precision constraint. The synergies appeared more variable during the precision condition (figure 5) and the weighting coefficients were ‘shared’ between participants during the precision condition, i.e. the wrist joint moment for participant#2 was loaded with the wrist, elbow, and shoulder joint moment of participant#1 in the first conjoint synergy (figure 7*a*). These results suggest that although the coordination was more variable during the precision condition, more covariation occurred across the joint moments of the two participants. These results show that the collaboration during the precision task required disorganization of the spontaneous coordination adopted by the participants when no precision constraint was present. The change in posture between CC and PC might partly explain this observation. The VAF for the conjoint analysis tended to be lower during the precision condition (i.e. *p* = 0.06), also suggesting a more variable coordination pattern between the joint moments of the two participants. These results suggest that coordinated action between two participants does not necessarily require a similar coordination pattern for each of them, i.e. similar dynamic synergies, and that, in our experiments, a new coordination pattern emerged between the two participants (i.e. different joint synergies), with more co-variation between their joint moments.

## 5. Conclusion

This study highlights the fact that when a dyad of individuals collectively transports a load and performs a second task (requiring accuracy and precision), the displacement of the CoM of the whole system (PACS) is affected, inducing a less efficient pendulum-like behaviour. In both conditions, the individuals coordinated their forces to move in the vertical and forward direction without adopting a common strategy in the left-right direction. We also observed that the participants changed their trunk orientation and their behaviour to control the displacement of the ball inside the target. Furthermore, the visual feedback permitted the dyad to coordinate forces and movements in order to better control the position of the ball. However, the kinetic synergy analysis showed that participants altered the structure of their own synergies in PC to adopt a coordination that was dissimilar between participants but in which their wrist joint moments co-varied more, also leading to a lower cost at the upper limbs level during the precision task. These results could be of interest for people working in ergonomics and could find potential developments in robotics (e.g. human–robot interactions), and in the rehabilitation domain, for example, when several caregivers in healthcare establishments have to move a patient. Synergies are organized not only at the individual level but also at the level of the group, however linking two individuals need some constraints (load, precision). Future studies should investigate more deeply into what kind of individual predisposition and what kind of constraints favour the emergence of collective synergies.

## Data Availability

The dataset used as supplementary material mentioned in the manuscript is available on the repository Dryad (https://doi.org/10.5061/dryad.kprr4xh5n) [47] and on figshare (https://figshare.com/articles/dataset/Biomechanical_effects_of_the_addition_of_a_precision_constraint_on_a_collective_load_carriage_task/20456460).
